# Supplementary figures and images for: Modeling transmission dynamics and effectiveness of worker screening programs for SARS-CoV-2 in pork processing plants
Source: PLoS One. 2021 Sep 2;16(9):e0249143. doi: 10.1371/journal.pone.0249143 (PMC8412359; doi:10.1371/journal.pone.0249143)

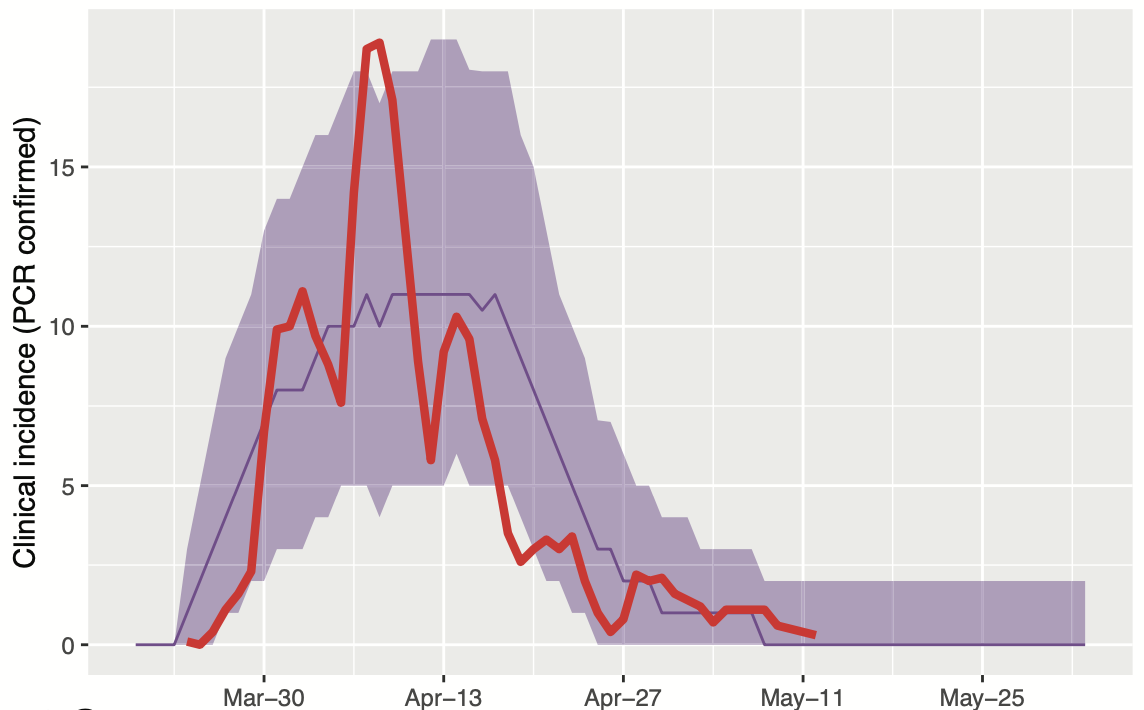

Supplement: S1 Fig — (PNG) [file pone.0249143.s001.png]
