# Supplementary material for: Modeling transmission dynamics and effectiveness of worker screening programs for SARS-CoV-2 in pork processing plants
Source: PLoS One. 2021 Sep 2;16(9):e0249143. doi: 10.1371/journal.pone.0249143 (PMC8412359; doi:10.1371/journal.pone.0249143)
Supplement: S1 Appendix — (PDF) [file pone.0249143.s002.pdf]

Supplementary information: Appendix S1

Modeling transmission dynamics and effectiveness of worker screening programs for SARS-CoV-2 in pork processing plants

Supplementary text A. Incidence data

For Plants A and B, individual confirmed cases were dated to either the test result date or the absentee date, whichever was earliest. These two dates were a median of 0 days apart (IQR: -0.75 – 2 days). For Plant C, tests were administered on the first day of symptoms, and all close contacts home-quarantined (with full pay) at the same time.

Supplementary text B. Sensitivity Analysis

We performed a sensitivity analysis for the stochastic model using Latin hypercube sampling and random forest analyses, which is a common approach used for global sensitivity analyses in simulation modelling [1-4]. Random forest is an ensemble machine learning method that is based on the consensus of hundreds of randomized decision trees [5]. Unlike regression approaches, random forests can handle more complex data with numerous interacting and multicollinear variables with non-linear effects on the outcome [6,7], making them a highly suitable approach for understanding the marginal contribution of variation in each parameter on the outcome after controlling for the effects of all other parameters. Here, the sensitivity analysis not only included the rate parameters from the deterministic model, but also parameters that control the frequency, diagnostic sensitivity, and delays in results for company-initiated PCR testing and daily temperature screening (Table 2). Briefly, we generated 1,000 parameter sets sampled from a Latin hypercube, and simulated 1,000 epidemics for each set (population size=1000, duration of simulation = 365 days). The median number of cumulative infections was summarized for each set, and this value was used as the outcome in the random forest. Parameter values were included in the random forest as explanatory variables to explain variation in the outcome [1,8]. To assess the overall performance of the model, the percent of variance explained by the random forest was calculated. Variable importance was assessed by calculating the percent increase in mean standard error (MSE) of predictions when x is permuted relative to y, with larger changes in MSE indicating that the variable is an important predictor of the outcome. Partial dependence plots were constructed to assess the marginal effect of model parameters on model outputs after accounting for the effects of all other parameters.

Supplementary text C. Accessory methods for estimating R

For the EG method, R was inferred from the early phase of the outbreak, during which time the epidemic curve approximates exponential growth. The EG method combines the observed per capita change in number of new cases per day with an assumed generation time to estimate the population's R [9,10]. Second, we applied the maximum-likelihood (ML) method proposed in [9,11], which is built on the assumption that the number of secondary cases for each infected individual follows a Poisson distribution with a mean R that can be estimated though a likelihood-based approach. Generation time was assumed to follow a gamma distribution with a mean of six days and standard deviation of one day [12-14]. The "early phase" time period (identified via built-in functions in the *R0* package as the period where cases approximated exponential growth) used for the EG and ML methods is shown in Supplementary Figure S2.

Supplementary Table A. Parameter definitions, symbols and types. Plausible range indicates the reasonable range of values for each parameter. (*) indicate parameters that were only used for the stochastic model sensitivity analysis, whereas non-starred parameters were used both for deterministic model calibration and stochastic sensitivity analysis. Calibrated values show the most likely (median) values from model calibration for each plant's data, with the interquartile range shown in parenthesis.

**Definition**		**Type**	**Plausible range**	**Plant A**	**Plant B**	**Plant C**
Reproduction number	R	disease	1.0 – 8.0 [15-25]	1.7 (1.2 – 2.8)	3.2 (2.6 – 4.6)	2.1 (1.7 - 3.2)
Relative infectious of undetected "carriers"	R_c	disease	0.05 – 1.0 [26-31]	0.36 (0.22-0.4)	0.26 (0.13-0.46)	0.46 (0.21 – 0.72)
background transmission rate(b)	b	disease	0.0001- 0.01	0.005 (0.003-0.005)	0.009 (0.008-0.01)	0.007 (0.005 – 0.008)
Percent undetected (q)	q	disease	0.1 - .95 [12,26,30-36]	0.77 (.69-.81)	0.51 (0.46 – 0.59)	0.32 (0.23 – 0.41)
Latent period	$1/\lambda_1$	disease	3 – 9 [17,37,38]	5.4 (4.3 – 5.7)	4.5 (4.0 – 5.0)	6.1 (4.4 – 7.6)
Pre-symptomatic period	$1/\lambda_2$	disease	1-2.5 [13,39]	1.5 (1.3 – 1.9)	1.8 (1.5 – 2.2)	1.7 (1.3 – 2.1)
Clinical (infectious) period	$1/\gamma$	disease	2.5 – 11 [13]	7.95 (5.5 – 8.8)	5.5 (5.1 – 9.0)	7.7 (5.2 – 9.5)
Prop. people that go to work when ill	z	Worker behavior	.05 – 0.5 [40]	0.23 (0.17 – 0.34)	0.26 (0.13 – 0.36)	0.23 (0.15 – 0.34)
At-home isolation period (default = 10 days)	$1/h$	Worker behavior	7 – 21*			
Sensitivity of daily temperature screening (detection of I_c)	d_{temp}	Temp. screening	0.6 – 0.9* [41,42]			
Sensitivity of PCR testing (detection of I_c, I_c, C)	d_{pcr}	PCR testing	0.85 – 1.0* [43,44]			
Frequency of company-initiated PCR testing (interval in days)		PCR testing	7 – 28*			
Start day of company-initiated PCR testing		PCR testing	1 – 183*			
Delay in results for PCR test		PCR testing	0 – 7*			

Supplementary Table B. Results of antibody testing at Plant B.

Date	IgM-IgG	No. tested	Prop. positive	95-CI: Lower bound	95-CI: Upper bound
**4/17/20**	Neg-neg	66	0.75757576	0.65418418	0.86096734
**4/17/20**	Neg-pos	66	0.01515152	-0.0143196	0.04462265
**4/17/20**	Pos-neg	66	0.04545455	-0.0047995	0.0957086
**4/17/20**	Pos-pos	66	0.18181818	0.08876576	0.2748706
**4/17/20**	IgG-pos	66	0.1969697	0.10101864	0.29292076
**4/24/20**	Neg-neg	192	0.546875	0.47646108	0.61728892
**4/24/20**	Neg-pos	192	0.015625	-0.0019177	0.03316767
**4/24/20**	Pos-neg	192	0.05208333	0.02065366	0.08351301
**4/24/20**	Pos-pos	192	0.38541667	0.31657346	0.45425987
**4/24/20**	IgG-pos	192	0.40104167	0.33171529	0.47036805
**5/1/20**	Neg-neg	133	0.65413534	0.57329704	0.73497363
**5/1/20**	Neg-pos	133	0.02255639	-0.0026791	0.04779183
**5/1/20**	Pos-neg	133	0	0	0
**5/1/20**	Pos-pos	133	0.32330827	0.24381432	0.40280222
**5/1/20**	IgG-pos	133	0.34586466	0.26502637	0.42670296
**5/8/20**	Neg-neg	59	0.57627119	0.45017919	0.70236318
**5/8/20**	Neg-pos	59	0.01694915	-0.0159885	0.04988676
**5/8/20**	Pos-neg	59	0.01694915	-0.0159885	0.04988676
**5/8/20**	Pos-pos	59	0.38983051	0.26538099	0.51428003
**5/8/20**	IgG-pos	59	0.40677966	0.28143158	0.53212774
**5/12/20**	Neg-neg	22	0.59090909	0.38545491	0.79636327
**5/12/20**	Pos-pos	22	0.40909091	0.20363673	0.61454509
**5/12/20**	IgG-pos	22	0.40909091	0.20363673	0.61454509

Supplementary Table C. Key indicators from observed data, goodness-of-fit criteria used for rejection sampling, and the number of parameter sets that met criteria and were used to define the calibrated parameter values.

	**A**	**B**	**C**
Key indicators from observed data	• Time.10H: Day in which a cumulative of 10+ people were at-home sick (i.e., self-reports), must be within 18 days of first case<br>• Time.15H: Day in which a cumulative of 15+ people were at-home sick, which was observed to occur 6 days after Time.10H (4-12 days)<br>• Proportion recovered at Time.15H is in the 95-CI of the IgG-positive rate: 3.6 - 9.2%	• Time.20H: Day in which a cumulative of 20+ people were at-home sick (i.e., self-reports)<br>• Time.100H: Day in which a cumulative of 100+ people were at-home sick, which was observed to occur 7 days after Time.20H<br>• Time.200H: Day in which a cumulative of 200+ people were at-home sick, which was observed to occur 9 days after Time.100H<br>• Time.20R: Day in which 20% of workers are recovered (presumed IgG+), observed to occur same week as 200H<br>• R.3wk_post20: Percent recovered two weeks post time.20R, which was observed to be 40±13%	• Time.5H: Day in which cumulative of 5+ people were at-home sick<br>• Time.50H: Day in which cumulative of 50+ people were at-home sick, observed to be 14 days (±4 from time.5H)<br>• Time100.H: Day in which cumulative of 50+ people were at-home sick, observed to be 14 days from time.50H (±4 days)<br>• Time.90H: Day in which cumulative of 90+ people were at-home sick<br>• R.at.time.90H: Percent recovered (presumed AB+) was observed to be 16.6% (12.9-20.3%) at time.90H
"Goodness-of-fit" criteria	• Time.10H < 18<br>• Time.10H+4 days < Time.15H < Time.10H + 12<br>• 3.6% < R at Time.15H < 9.2%	• 2 < time.100H < 10<br>• Time.100H+5 < time.200H < Time.100H + 14<br>• Time.20R < time 200H +5<br>• 27% < R.3wks_post20 < 53%	• Time.5H +10 < time.50H < time.5H+18<br>• Time.50H + 10 < time.100H <- time.50H + 18<br>• 12.9% < R.at.time.90H < 20.3$
Parameter sets meeting criteria	18/10,000	17/10,000	255/20,000

Supplementary Table D. Number of Plant B workers positive and negative to IgG and IgM according to self-reported PCR status.

	IgM/IgG				
**Self-reported PCR test:**	**-/-**	**-/+**	**+/-**	**+/+**	**Total**
Not Tested	278	7	11	123	419
Negative COVID19 test	8	0	0	3	11
Positive COVID19 test	2	1	3	28	34
Presumptive Positive	1	0	0	7	8
**Total**	**289**	**8**	**14**	**161**	**472**

Supplementary Table E. Estimated *R*'s for Plants A-C based on four methods.

**Method**	**A**	**B**	**C**
Exponential growth (EG)	2.3 (0.8 – 7.8)	3.8 (2.9 – 5.2)	2.8 (1.9 – 4.5)
Maximum-likelihood (ML)	2.7 (0.8 – 6.2)	3.0 (2.2 – 3.8)	3.2 (2.0 – 4.9)
Time-dependent R (TD-R, week 1)	2.5 (2.0 – 3.0)	4.4 (3.5 – 5.5)	2.8 (2.0 – 3.5)
Model	1.7 (1.2 – 2.8)	3.2 (2.6 – 4.6)	2.1 (1.7 – 3.2)

Supplementary Figure A. The time period during which the epidemic curve best approximated exponential growth is shown between the blue-hashed lines for Plants A, B, and C. This period was identified through the built-in default settings utilized by the *R0* package.

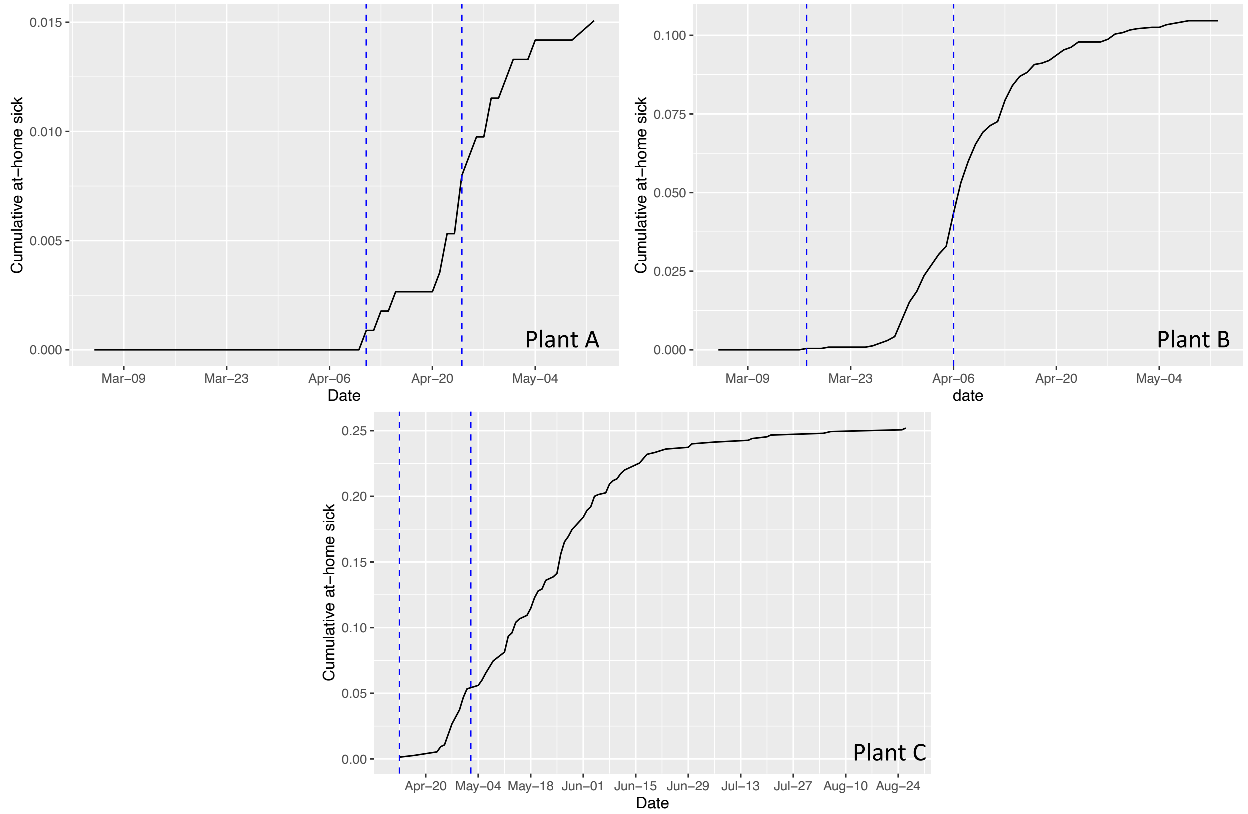

Supplementary Figure B. Top: Cumulative proportion of at-home sick workers predicted by the tuned models (black) compared to the observed incidence data (red). Bottom: Cumulative proportion infected predicted by the tuned models (black line) compared to the observed antibody testing data (circles). Data prior the blue dashed line (date of conclusion of company-initiated testing) were used for initial model.

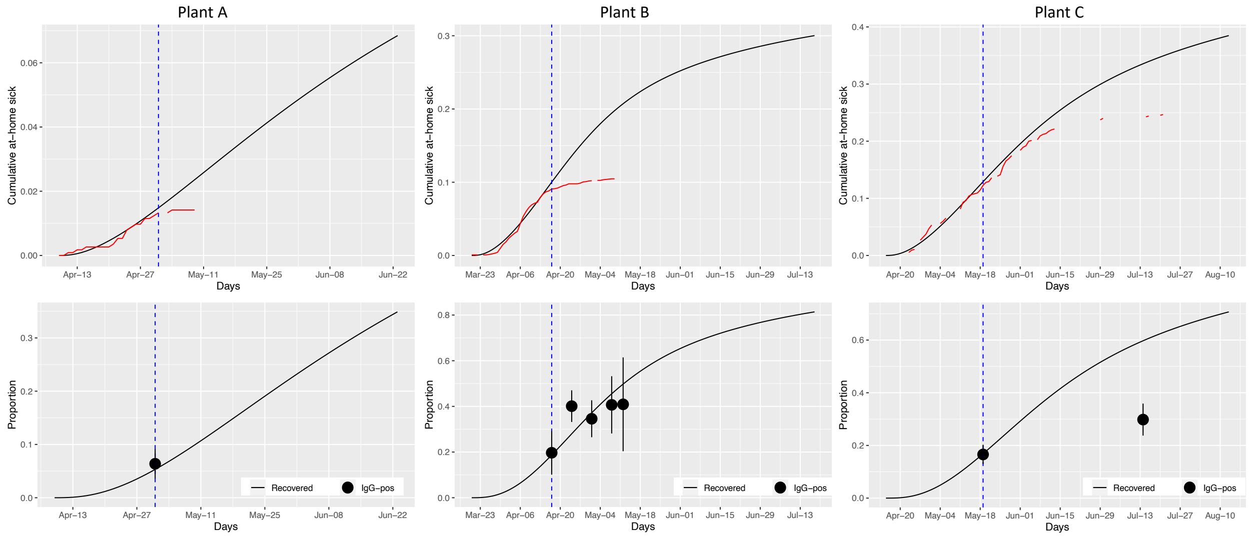

Supplementary Figure C. Number of transmission events due to workplace-based transmission (red) versus community transmission (black).

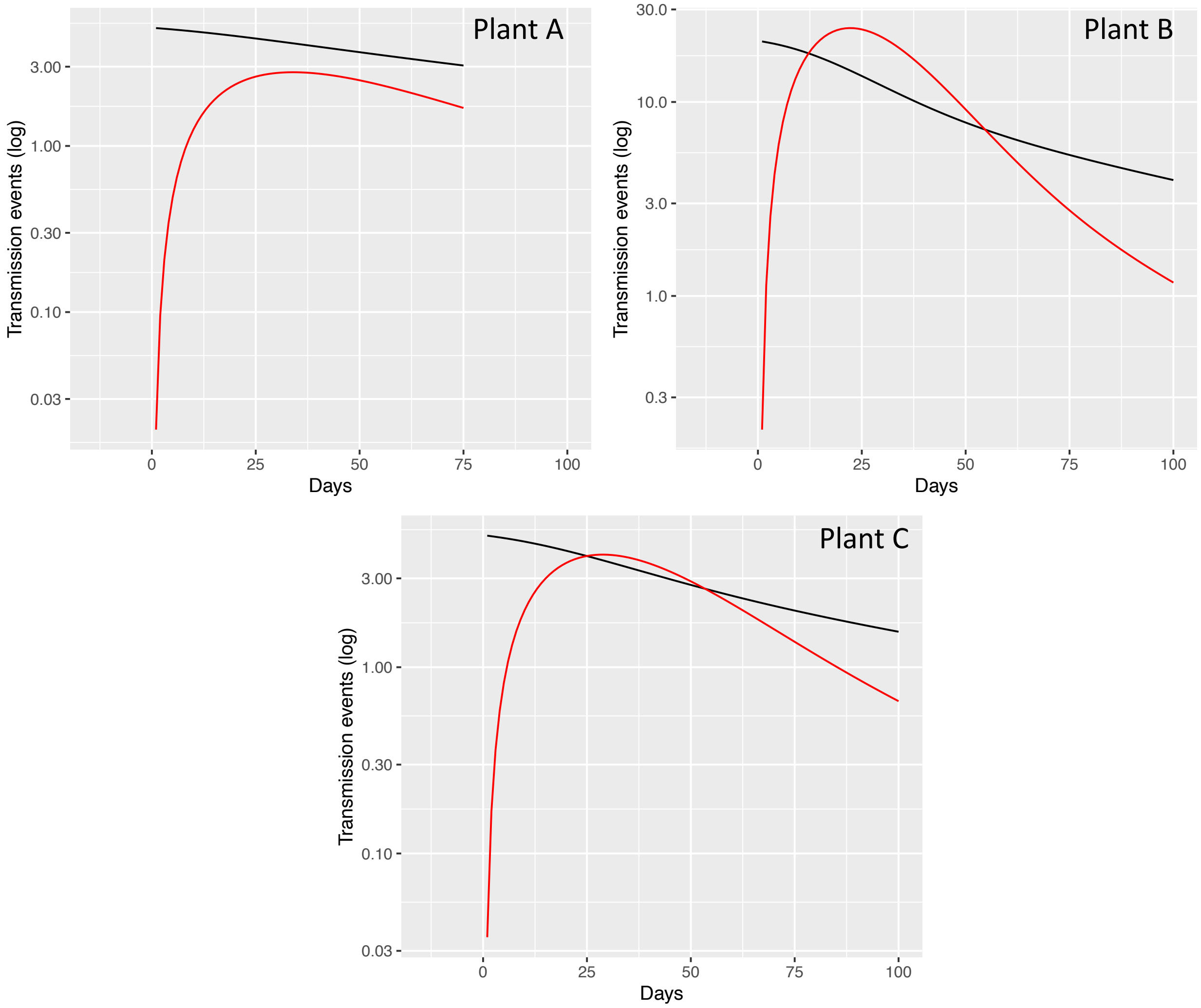

Supplementary Figure D. Partial dependence plots showing the marginal effect of varying each parameter on the cumulative number of infections.

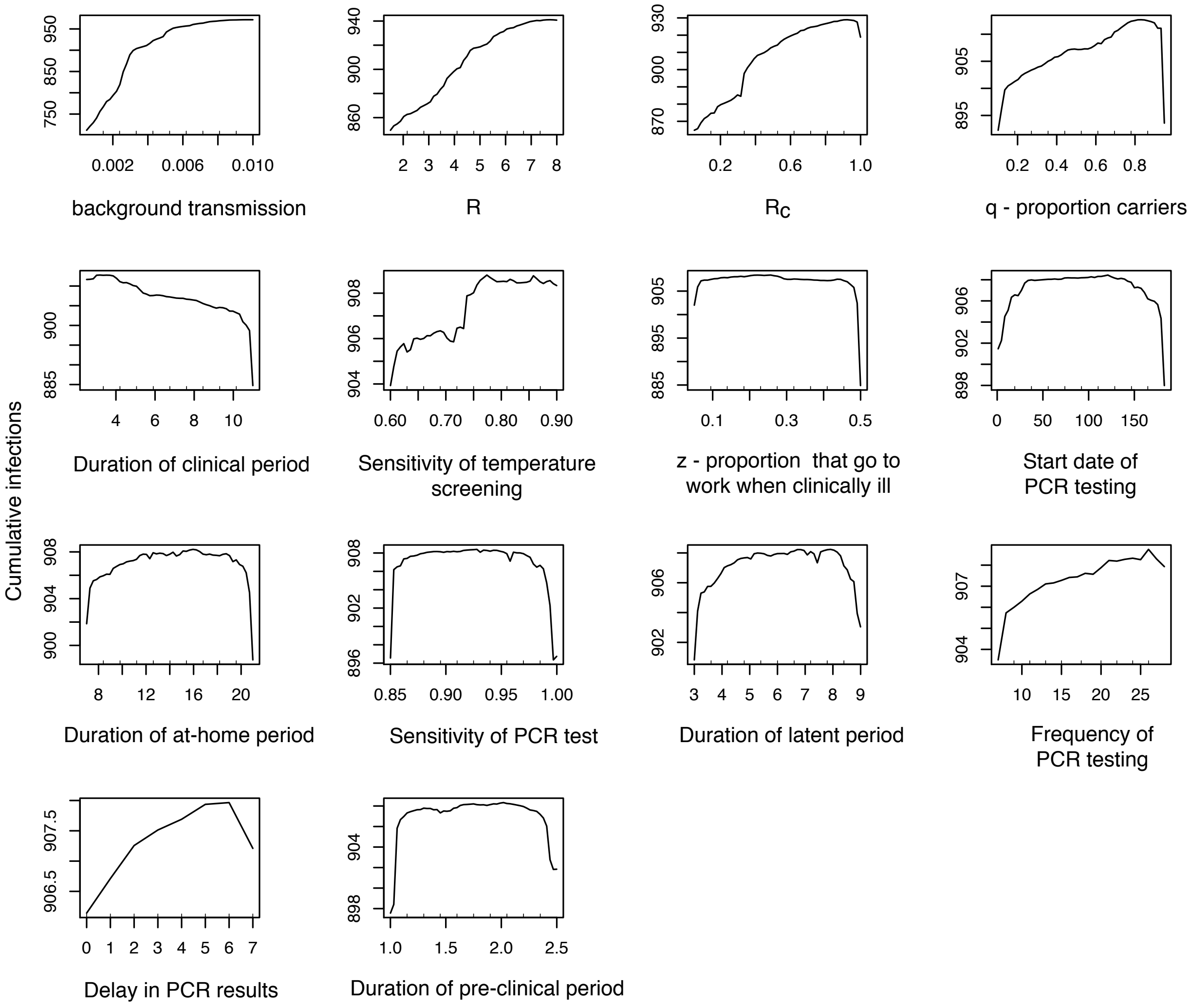

Supplementary Figure E. Comparison of R estimates using different estimation techniques. Colors indicate each technique: EG: Exponential growth; ML: Maximum likelihood; TD: R-TD during the first week; Model: Estimate from the tuned compartmental model. Shape indicates the assumed generation time.

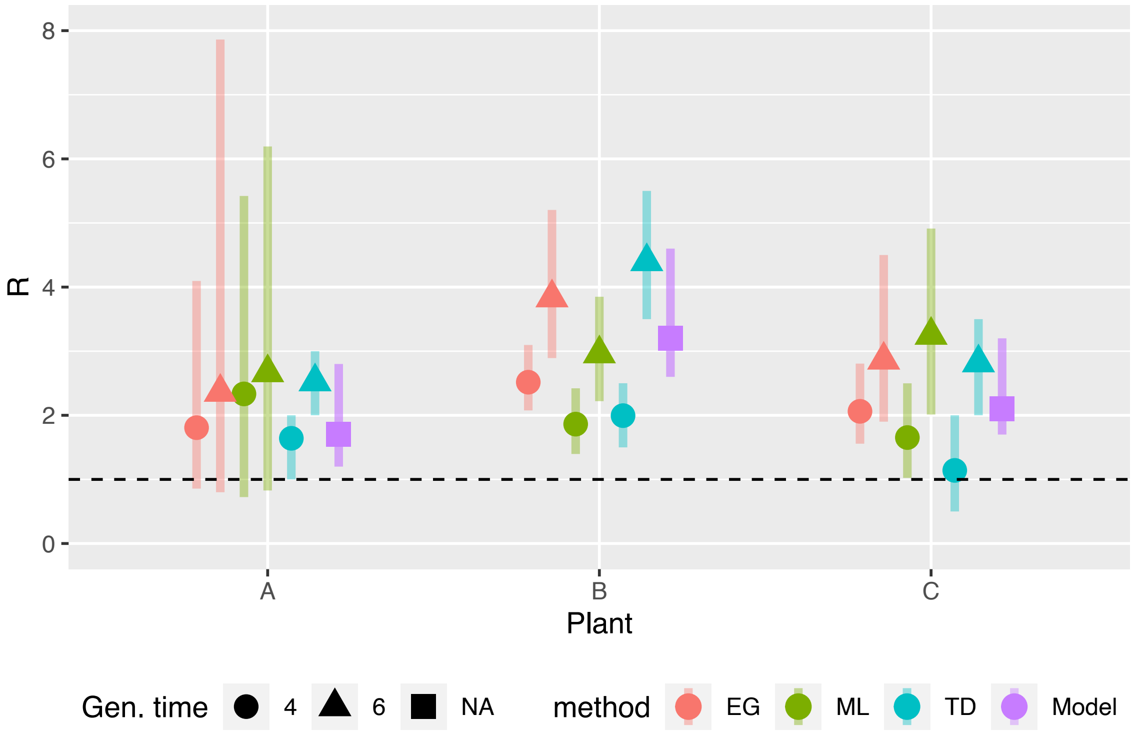

Supplementary Figure F. Time-dependent R estimated at a weekly interval for each plant, assuming generation time of four days (top) and six days (bottom).

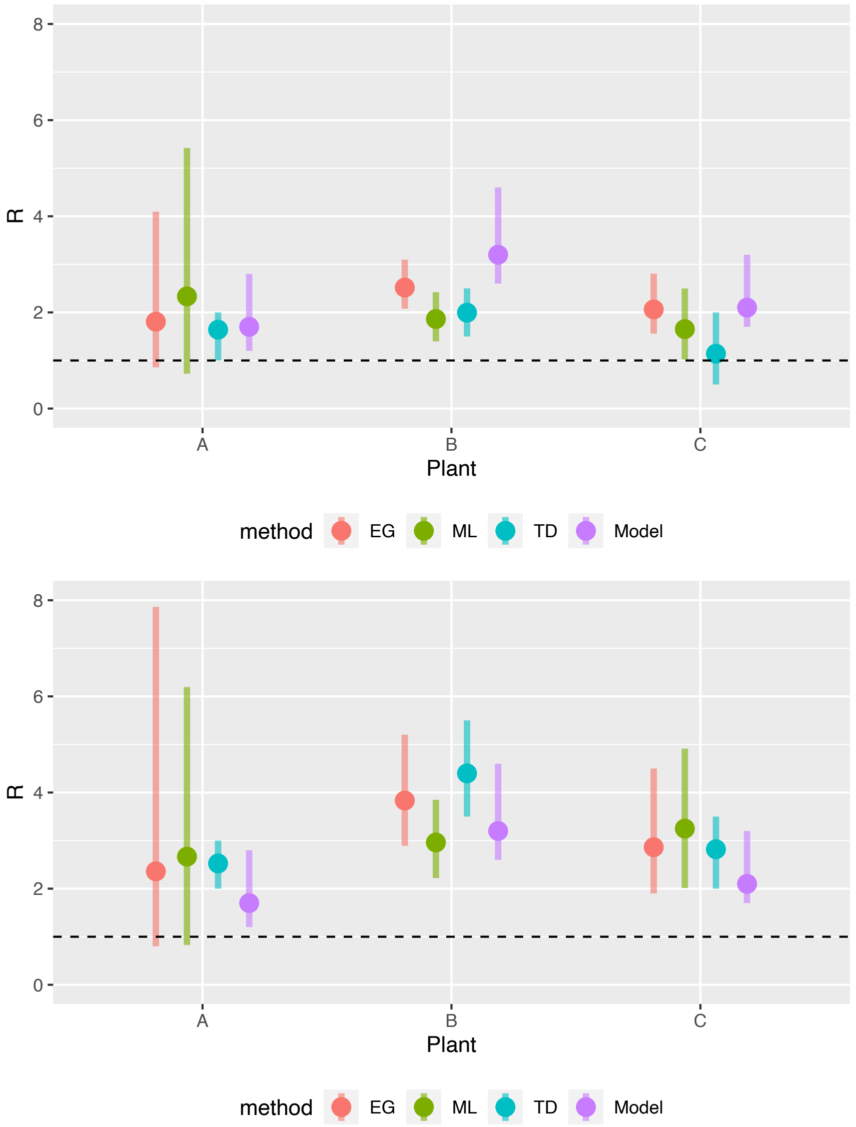

Supplementary Figure G. Cumulative number of additional infections. Results are stratified according to stage at which PCR-screening is implemented (Prevention, Early, Peak, Post, 35% Immune), workforce size (n = 100, 1000, 2500), and high/low workforce *R* and background transmission rates. Each cell label shows the absolute number of additional infections. Colored shading indicates the cumulative proportion of the workforce that is infected.

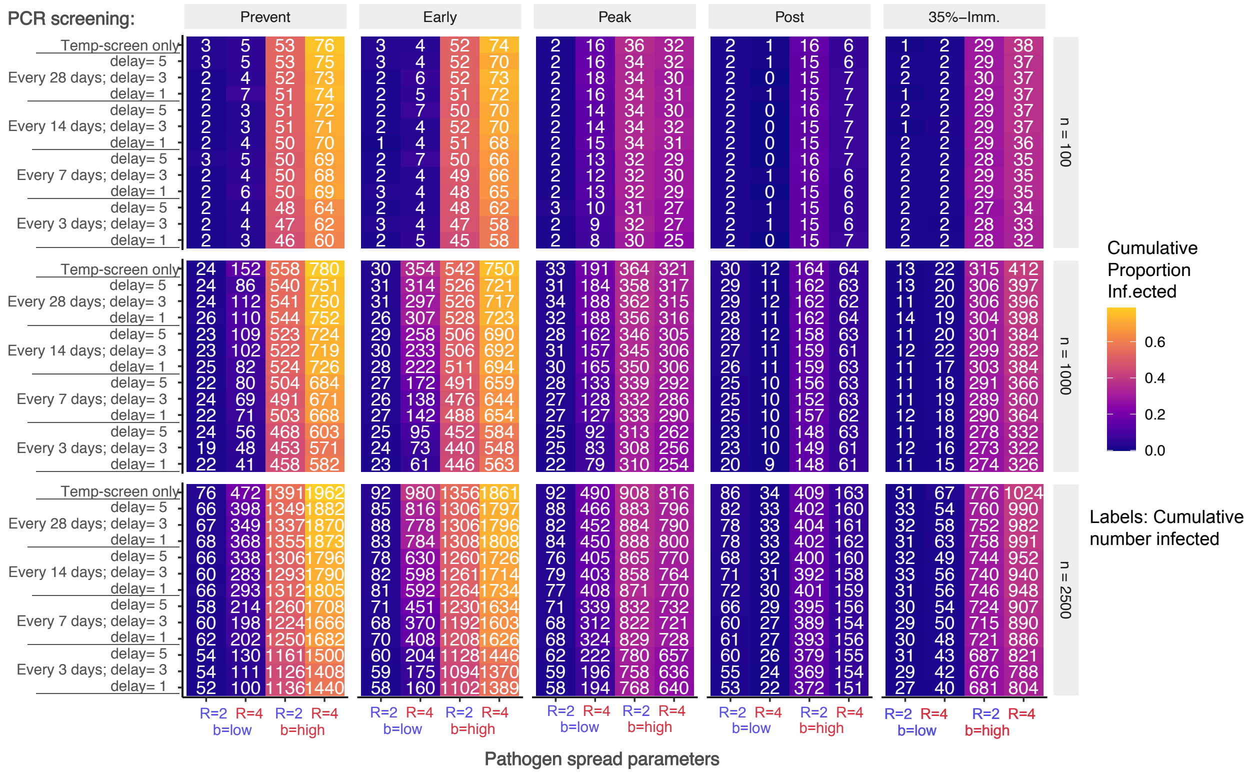

Supplementary Figure H. Cumulative number of additional clinical cases. Results are stratified according to stage at which PCR-screening is implemented (Prevention, Early, Peak, Post, 35% Immune), workforce size (n = 100, 1000, 2500), and high/low workforce *R* and background transmission rates. Each cell label shows the absolute number of additional clinical cases. Colored shading indicates the cumulative proportion of the workforce that experiences clinical disease.

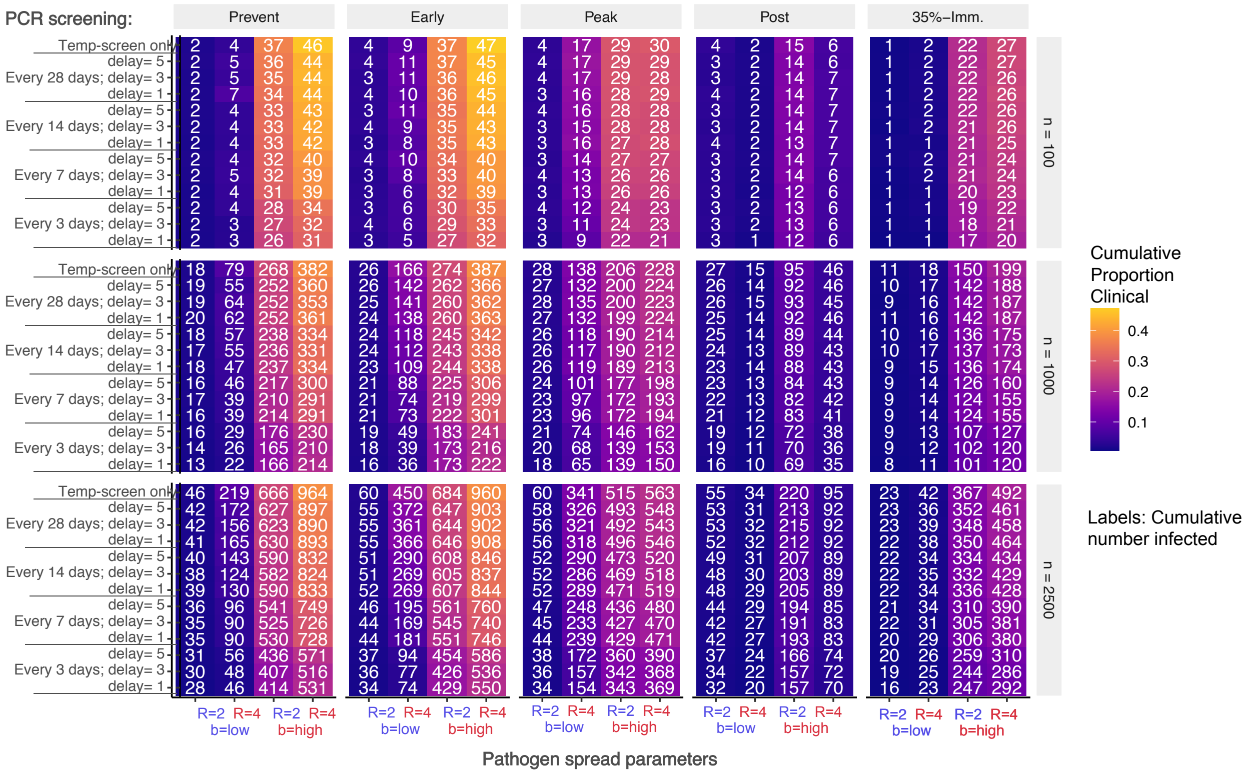

Supplementary Figure I. Proportion of infections due to workplace transmission. Results are stratified according to stage at which PCR-screening is implemented (Prevention, Early, Peak, Post, 35% Immune), workforce size (n = 100, 1000, 2500), and high/low workforce *R* and background transmission rates. Each cell label shows the cumulative number of additional workplace transmission events. Colored shading indicates the proportion of all transmission events that were attributed to the workplace.

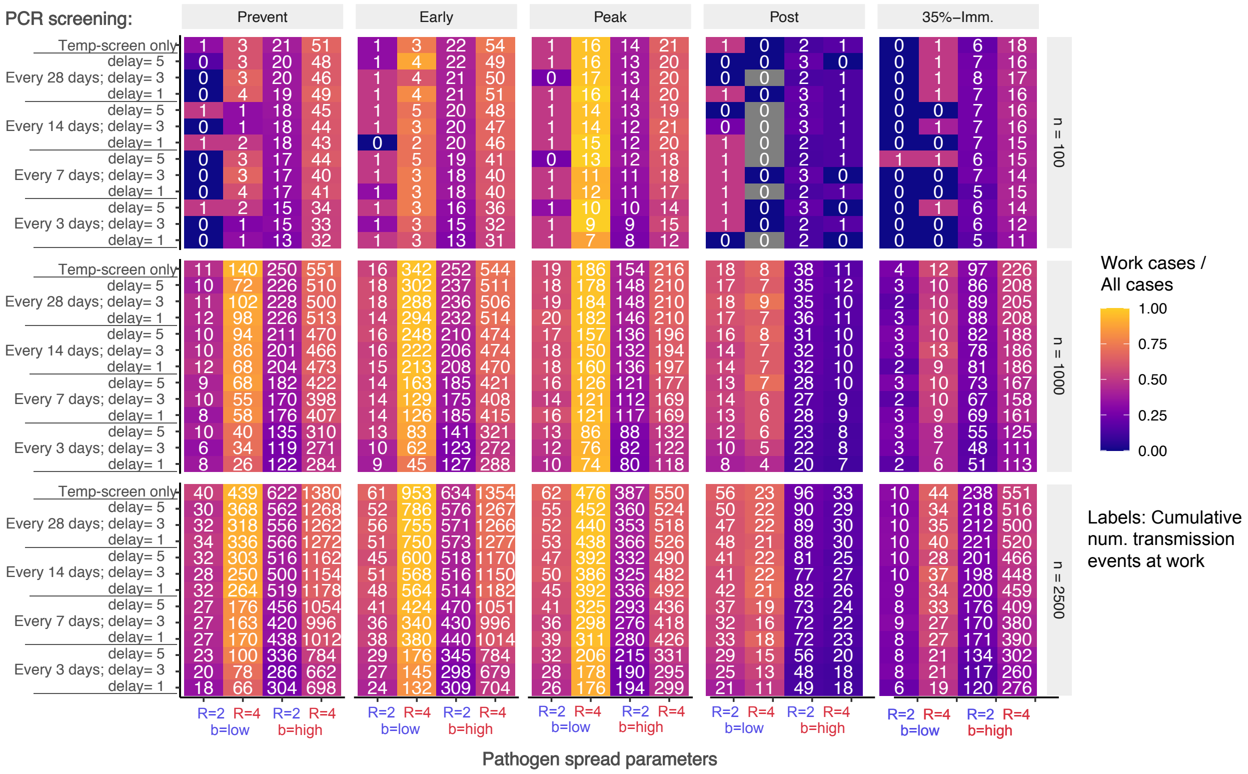

Supplementary Figure J. Number of infections detected via PCR-screening. Results are stratified according to stage at which PCR-screening is implemented (Prevention, Early, Peak, Post, 35% Immune), workforce size (n = 100, 1000, 2500), and high/low workforce *R* and background transmission rates. Each cell label shows the absolute number of infections detected via PCR-screening. Colored shading indicates the proportion of infections that were detected.

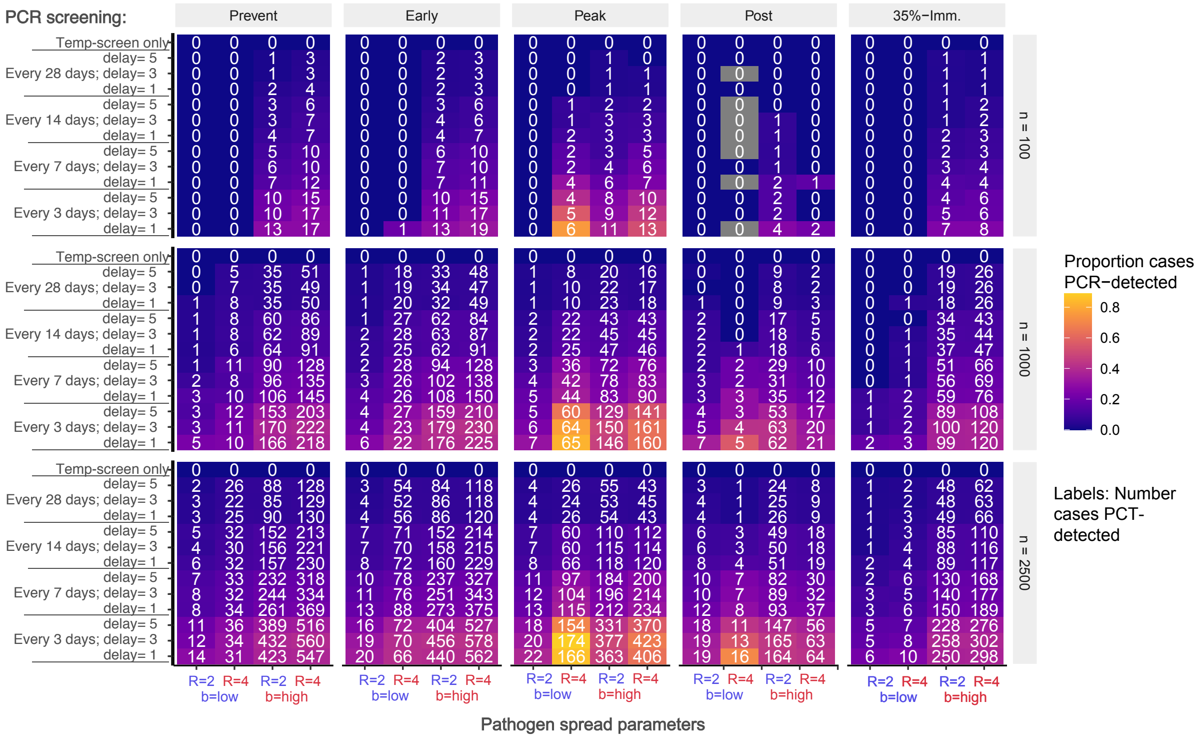

Supplementary Figure K. Number of expected clinical cases averted by PCR-screening relative to baseline of temperature screening alone. Results are stratified according to stage at which PCR-screening is implemented (Prevention, Early, Peak, Post, 35% Immune), workforce size (n = 100, 1000, 2500), and high/low workforce *R* and background transmission rates. Each cell label shows the absolute number of infections averted relative to the baseline (top row of each cell block). Colored shading indicates the number of cases averted.

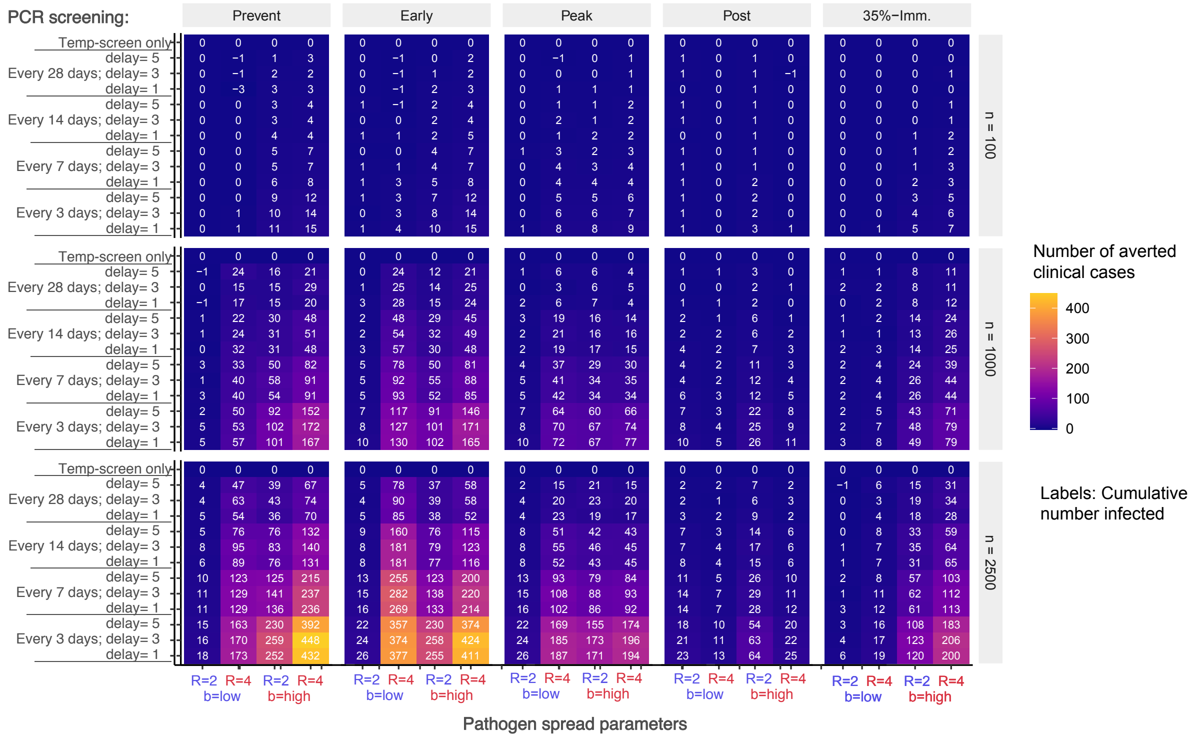

Supplementary Figure L. Proportion of expected clinical cases averted by PCR-screening relative to baseline of temperature screening alone when only 75% of workers participate in the screening program. Results are stratified according to stage at which PCR-screening is implemented (Prevention, Early, Peak, Post, 35% Immune), workforce size (n = 100, 1000, 2500), and high/low workforce *R* and background transmission rates. Absolute number of expected clinical cases under the baseline is labeled in the top line of each colored block, and each cell label shows the clinical cases relative to baseline ($Cases_{scenario}$ / $Cases_{baseline}$). Colored shading indicates the cumulative proportion of the workforce that experiences clinical disease.

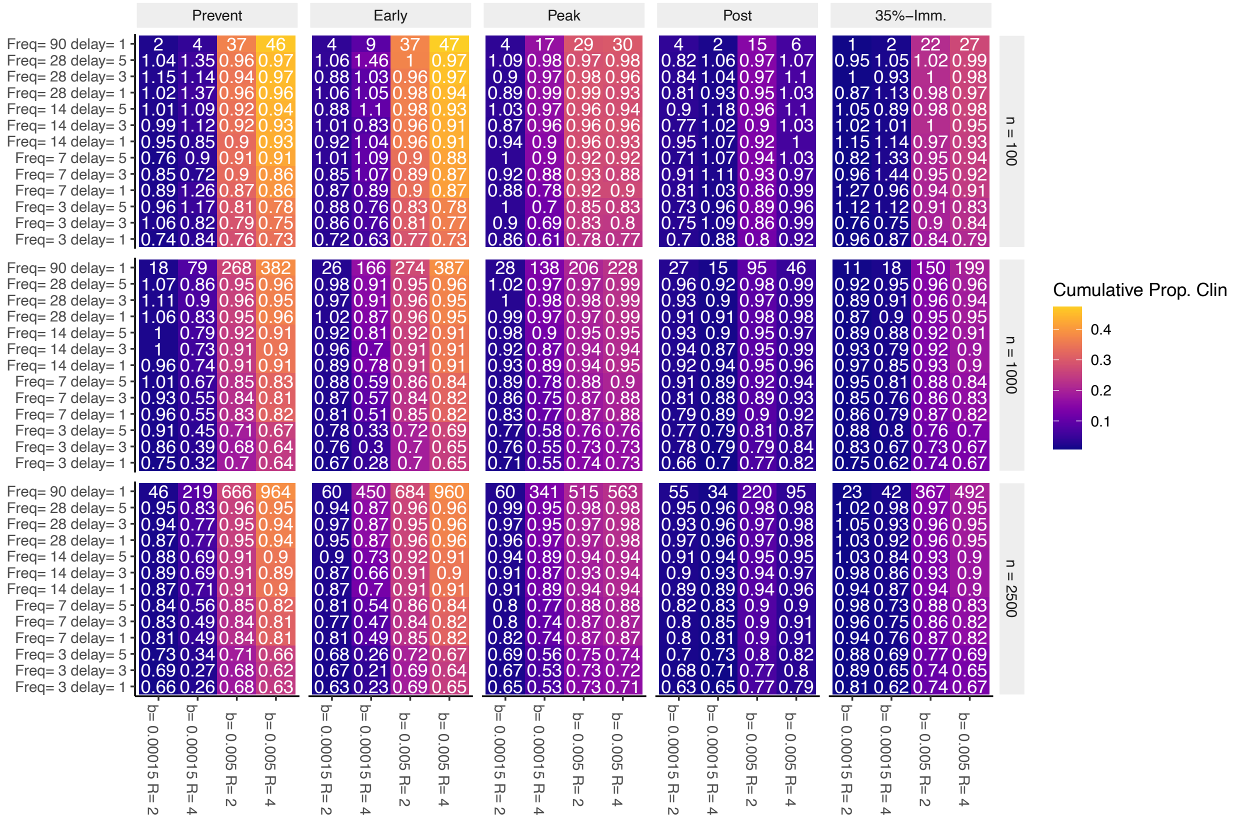

Supplementary Figure M. Cumulative number of additional infections when only 75% of workers participate in the screening program. Results are stratified according to stage at which PCR-screening is implemented (Prevention, Early, Peak, Post, 35% Immune), workforce size (n = 100, 1000, 2500), and high/low workforce *R* and background transmission rates. Each cell label shows the absolute number of additional infections. Colored shading indicates the cumulative proportion of the workforce that is infected.

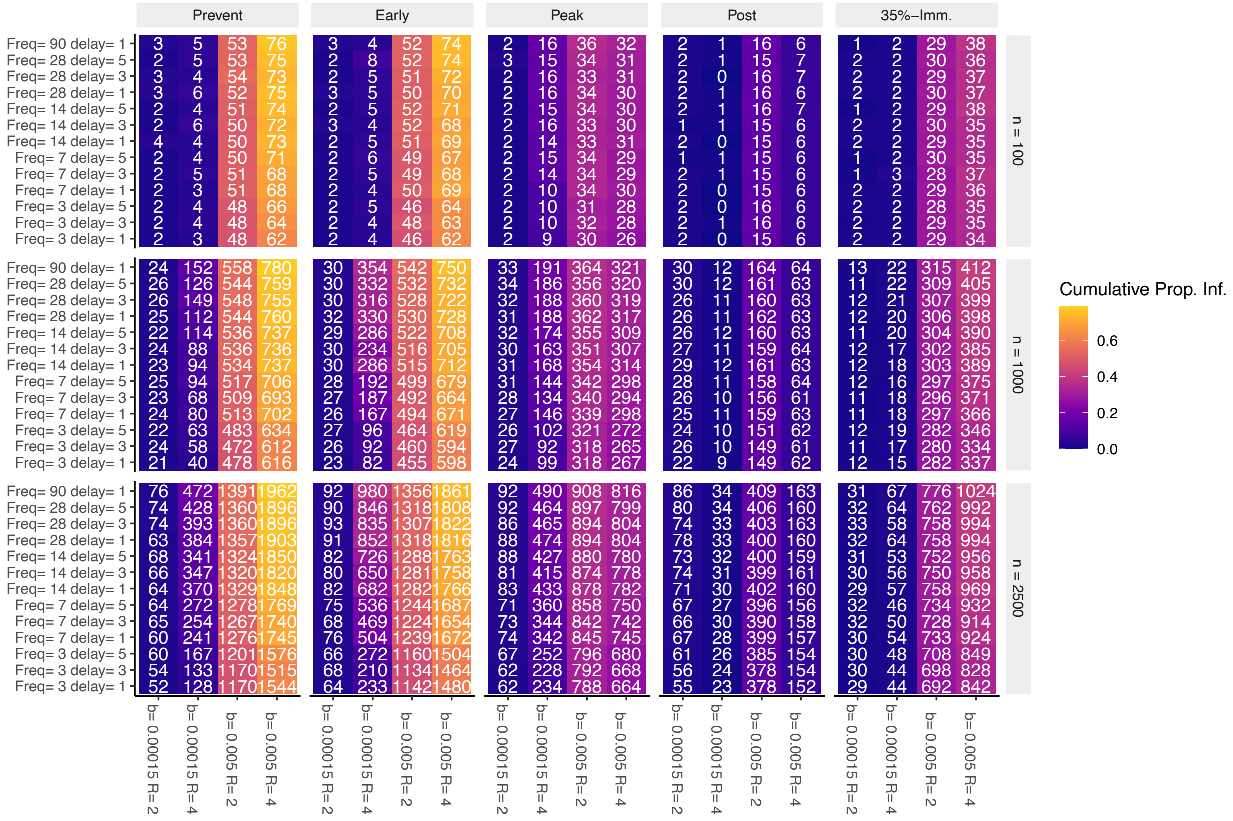

Supplementary Figure N. Cumulative number of additional clinical cases when only 75% of workers participate in the screening program. Results are stratified according to stage at which PCR-screening is implemented (Prevention, Early, Peak, Post, 35% Immune), workforce size (n = 100, 1000, 2500), and high/low workforce *R* and background transmission rates. Each cell label shows the absolute number of additional clinical cases. Colored shading indicates the cumulative proportion of the workforce that experiences clinical disease.

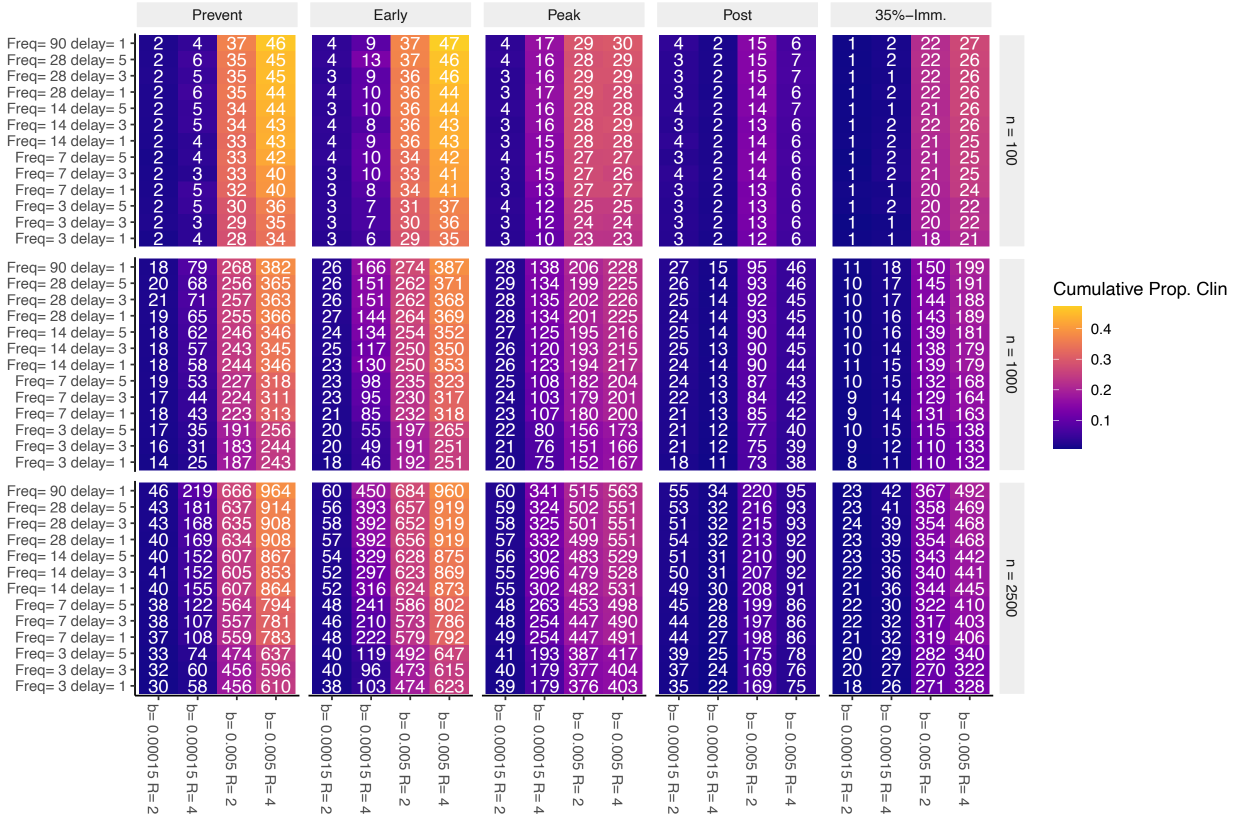

Supplementary Figure O. Proportion of infections due to workplace transmission when only 75% of workers participate in the screening program. Results are stratified according to stage at which PCR-screening is implemented (Prevention, Early, Peak, Post, 35% Immune), workforce size (n = 100, 1000, 2500), and high/low workforce *R* and background transmission rates. Each cell label shows the cumulative number of additional workplace transmission events. Colored shading indicates the proportion of all transmission events that were attributed to the workplace.

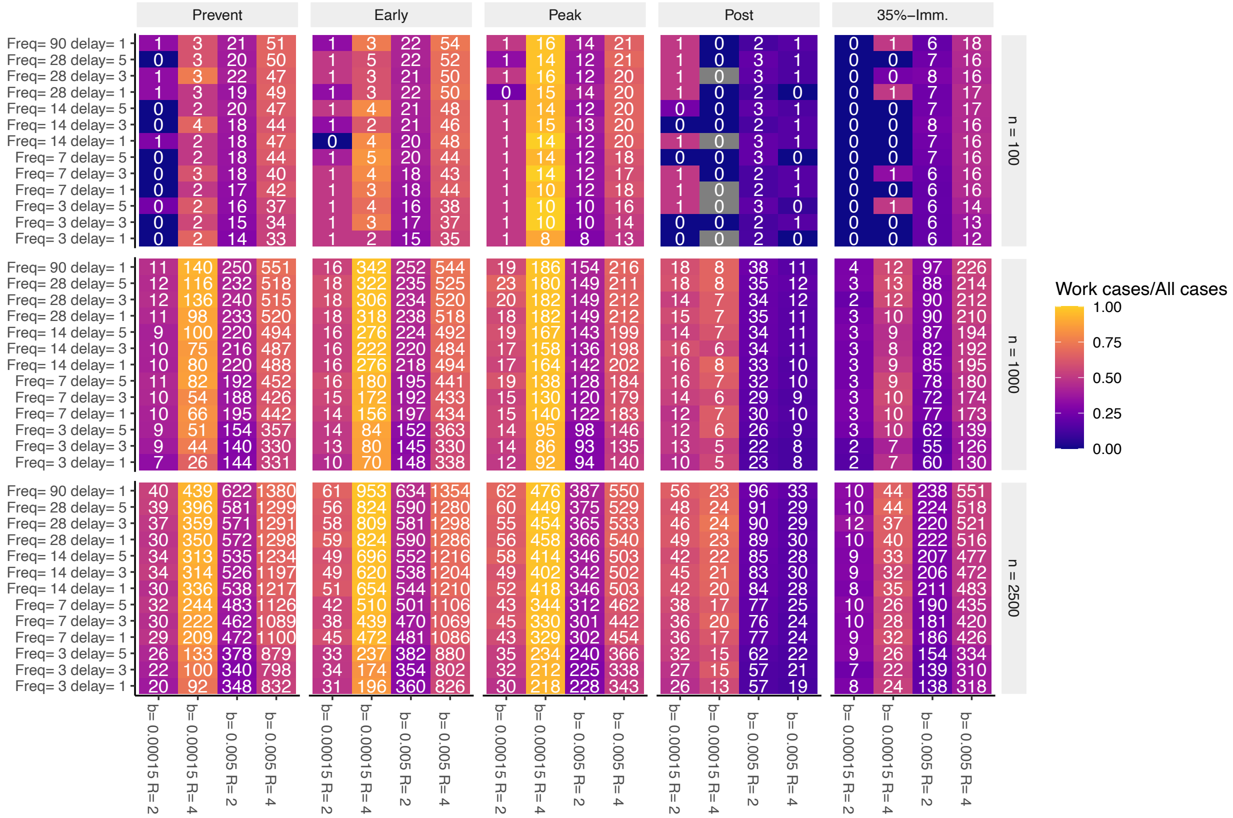

Supplementary Figure P. Number of infections detected via PCR-screening when only 75% of workers participate in the screening program. Results are stratified according to stage at which PCR-screening is implemented (Prevention, Early, Peak, Post, 35% Immune), workforce size (n = 100, 1000, 2500), and high/low workforce *R* and background transmission rates. Each cell label shows the absolute number of infections detected via PCR-screening. Colored shading indicates the proportion of infections that were detected.

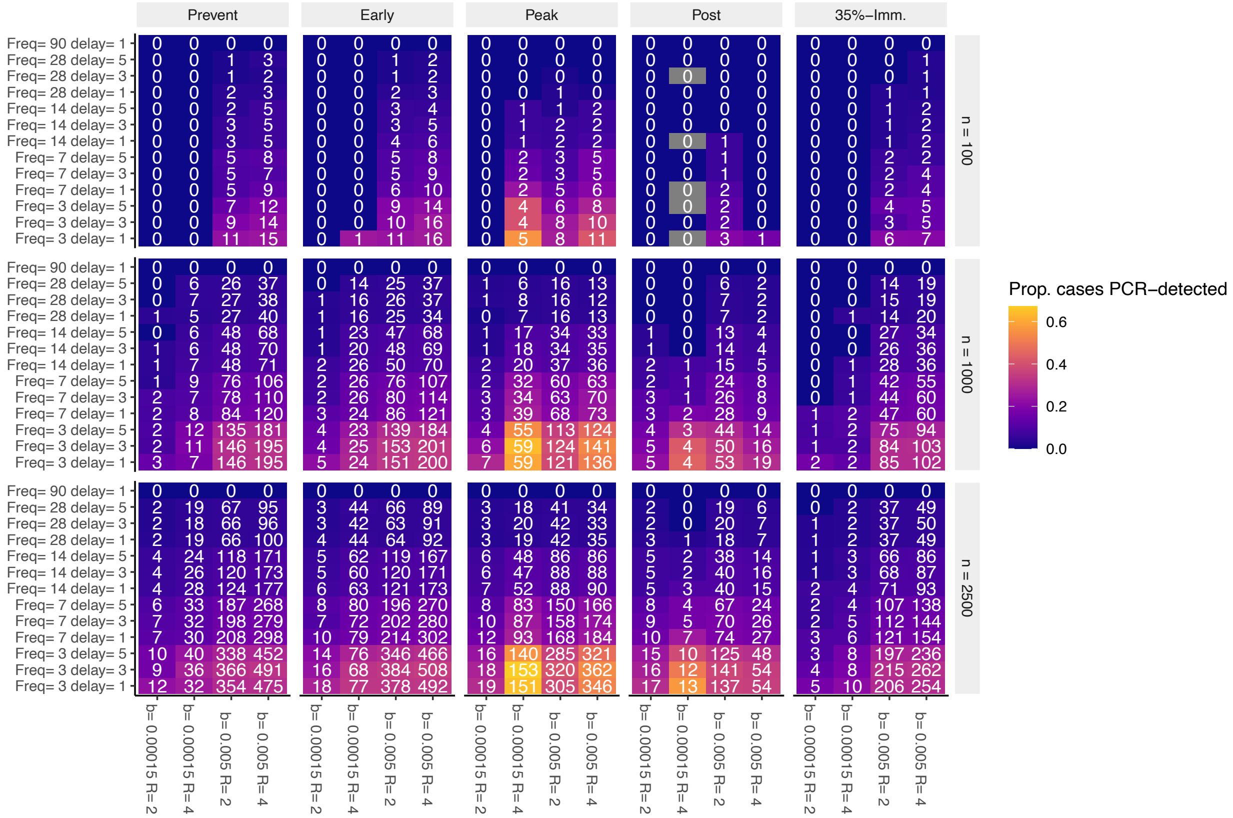

References

[1] VanderWaal, K., Perez, A.M., Torremorell, M., Morrison, R.B. & Craft, M. 2018 Role of animal movement and indirect contact among farms in transmission of porcine epidemic diarrhea virus. *Epidemics* 24, 67-75.
[2] Blower, S.M. & Dowlatabadi, H. 1994 Sensitivity and Uncertainty Analysis of Complex-Models of Disease Transmission - an Hiv Model, as an Example. *Int Stat Rev* 62, 229-243. (doi:Doi 10.2307/1403510).
[3] Legrand, J., Sanchez, A., Le Pont, F., Camacho, L. & Larouze, B. 2008 Modeling the Impact of Tuberculosis Control Strategies in Highly Endemic Overcrowded Prisons. *PLoS ONE* 3. (doi:ARTN e2100
10.1371/journal.pone.0002100).
[4] Wu, J.Y., Dhingra, R., Gambhir, M. & Remais, J.V. 2013 Sensitivity analysis of infectious disease models: methods, advances and their application. *J R Soc Interface* 10. (doi:ARTN 20121018
10.1098/rsif.2012.1018).
[5] Criminisi, A., Shotton, J. & Konukoglu, E. 2011 Decision forests for classification, regression, density estimation, manifold learning and semi-supervised learning. *Microsoft Research Cambridge, Tech. Rep. MSRTR-2011-114* 5, 12.
[6] Hultquist, C., Chen, G. & Zhao, K. 2014 A comparison of Gaussian process regression, random forests and support vector regression for burn severity assessment in diseased forests. *Remote Sensing Letters* 5, 723-732.
[7] Wang, B., Oldham, C. & Hipsey, M.R. 2016 Comparison of Machine Learning Techniques and Variables for Groundwater Dissolved Organic Nitrogen Prediction in an Urban Area. *Procedia Engineering* 154, 1176-1184.
[8] White, L.A., Torremorell, M. & Craft, M.E. 2017 Influenza A virus in swine breeding herds: Combination of vaccination and biosecurity practices can reduce likelihood of endemic piglet reservoir. *Prev Vet Med* 138, 55-69.
[9] Obadia, T., Haneef, R. & Boelle, P.Y. 2012 The R0 package: a toolbox to estimate reproduction numbers for epidemic outbreaks. *BMC Med Inform Decis Mak* 12, 147. (doi:10.1186/1472-6947-12-147).
[10] Wallinga, J. & Lipsitch, M. 2007 How generation intervals shape the relationship between growth rates and reproductive numbers. *Proceedings of the Royal Society B* 274, 599.
[11] White, L.F. & Pagano, M. 2008 A likelihood-based method for real-time estimation of the serial interval and reproductive number of an epidemic. *Stat. Med.* 27, 2999-3016. (doi:10.1002/sim.3136).
[12] Bi, Q., Wu, Y., Mei, S., Ye, C., Zou, X., Zhang, Z., Liu, X., Wei, L., Truelove, S.A., Zhang, T., Gao, W., Cheng, C., Tang, X., Qu, X., Wu, Y., Sun, B., Huang, S., Sun, Y., Zhang, J., Ma, T., Lessler, J. & Feng, T. 2020 Epidemiology and Transmission of COVID-19 in Shenzhen China: Analysis of 391 cases and 1,286 of their close contacts. *medXive* 2020.03.03.20028423.
[13] He, X., Lau, E.H.Y., Wu, P., Deng, X., Wang, J., Hao, X., Lau, Y.C., Wong, J.Y., Guan, Y., Tan, X., Mo, X., Chen, Y., Liao, B., Chen, W., Hu, F., Zhang, Q., Zhong, M., Wu, Y., Zhao, L., Zhang, F., Cowling, B.J., Li, F. & Leung, G.M. 2020 Temporal dynamics in viral shedding and transmissibility of COVID-19. *Nat. Med.* 26, 672-675. (doi:10.1038/s41591-020-0869-5).

[14] Koh, W.C., Naing, L., Rosledzana, M.A., Alikhan, M.F., Chaw, L., Griffith, M., Pastore, R. & Wong, W. 2020 What do we know about SARS-CoV-2 transmission? A systematic review and meta-analysis of the secondary attack rate, serial interval, and asymptomatic infection. *medXive* 2020.05.21.20108746.
[15] Sanche, S., Lin, Y.T., Xu, C., Romero-Severson, E., Hengartner, N. & Ke, R. 2020 High Contagiousness and Rapid Spread of Severe Acute Respiratory Syndrome Coronavirus 2. *Emerg Infect Dis* 26, 1470-1477. (doi:10.3201/eid2607.200282).
[16] Bar-On, Y.M., Flamholz, A., Phillips, R. & Milo, R. 2020 SARS-CoV-2 (COVID-19) by the numbers. *Elife* 9. (doi:10.7554/eLife.57309).
[17] Zhang, J., Litvinova, M., Wang, W., Wang, Y., Deng, X., Chen, X., Li, M., Zheng, W., Yi, L., Chen, X., Wu, Q., Liang, Y., Wang, X., Yang, J., Sun, K., Longini, I.M., Jr., Halloran, M.E., Wu, P., Cowling, B.J., Merler, S., Viboud, C., Vespignani, A., Ajelli, M. & Yu, H. 2020 Evolving epidemiology and transmission dynamics of coronavirus disease 2019 outside Hubei province, China: a descriptive and modelling study. *Lancet Infect. Dis.* 20, 793-802. (doi:10.1016/S1473-3099(20)30230-9).
[18] Li, Y., Wang, L.W., Peng, Z.H. & Shen, H.B. 2020 Basic reproduction number and predicted trends of coronavirus disease 2019 epidemic in the mainland of China. *Infect Dis Poverty* 9, 94. (doi:10.1186/s40249-020-00704-4).
[19] Zhang, S., Diao, M., Yu, W., Pei, L., Lin, Z. & Chen, D. 2020 Estimation of the reproductive number of novel coronavirus (COVID-19) and the probable outbreak size on the Diamond Princess cruise ship: A data-driven analysis. *Int J Infect Dis* 93, 201-204. (doi:10.1016/j.ijid.2020.02.033).
[20] Liu, Y., Gayle, A.A., Wilder-Smith, A. & Rocklöv, J. 2020 The reproductive number of COVID-19 is higher compared to SARS coronavirus. *Journal of Travel Medicine* 27.
[21] Muniz-Rodriguez, K., Fung, I.C., Ferdosi, S.R., Ofori, S.K., Lee, Y., Tariq, A. & Chowell, G. 2020 Severe Acute Respiratory Syndrome Coronavirus 2 Transmission Potential, Iran, 2020. *Emerg Infect Dis* 26, 1915-1917. (doi:10.3201/eid2608.200536).
[22] Caicedo-Ochoa, Y., Rebellon-Sanchez, D.E., Penaloza-Rallon, M., Cortes-Motta, H.F. & Mendez-Fandino, Y.R. 2020 Effective Reproductive Number estimation for initial stage of COVID-19 pandemic in Latin American Countries. *Int J Infect Dis* 95, 316-318. (doi:10.1016/j.ijid.2020.04.069).
[23] Li, C., Romagnani, P., von Brunn, A. & Anders, H.J. 2020 SARS-CoV-2 and Europe: timing of containment measures for outbreak control. *Infection* 48, 483-486. (doi:10.1007/s15010-020-01420-9).
[24] Liu, Y., Eggo, R.M. & Kucharski, A.J. 2020 Secondary attack rate and superspreading events for SARS-CoV-2. *Lancet* 395, e47. (doi:10.1016/S0140-6736(20)30462-1).
[25] Ives, A.R. & Bozzuto, C. 2020 State-by-State estimates of R0 at the start of COVID-19 outbreaks in the USA. *medXive* 2020.05.17.20104653. (doi:https://doi.org/10.1101/2020.05.17.20104653).
[26] Li, R., Pei, S., Chen, B., Song, Y., Zhang, T., Yang, W. & Shaman, J. 2020 Substantial undocumented infection facilitates the rapid dissemination of novel coronavirus (SARS-CoV-2). *Science* 368, 489-493. (doi:10.1126/science.abb3221).
[27] CDC. 2020 COVID-19 Pandemic Planning Scenarios. (United States Centers for Disease Control and Prevention.

[28] Gao, Z., Xu, Y., Sun, C., Wang, X., Guo, Y., Qiu, S. & Ma, K. 2020 A Systematic Review of Asymptomatic Infections with COVID-19. *J. Microbiol. Immunol. Infect.* (doi:10.1016/j.jmii.2020.05.001).
[29] Liu, Y., Yan, L.M., Wan, L., Xiang, T.X., Le, A., Liu, J.M., Peiris, M., Poon, L.L.M. & Zhang, W. 2020 Viral dynamics in mild and severe cases of COVID-19. *Lancet Infect. Dis.* 20, 656-657. (doi:10.1016/S1473-3099(20)30232-2).
[30] McMichael, T.M., Currie, D.W., Clark, S., Pogosjans, S., Kay, M., Schwartz, N.G., Lewis, J., Baer, A., Kawakami, V., Lukoff, M.D., Ferro, J., Brostrom-Smith, C., Rea, T.D., Sayre, M.R., Riedo, F.X., Russell, D., Hiatt, B., Montgomery, P., Rao, A.K., Chow, E.J., Tobolowsky, F., Hughes, M.J., Bardossy, A.C., Oakley, L.P., Jacobs, J.R., Stone, N.D., Reddy, S.C., Jernigan, J.A., Honein, M.A., Clark, T.A., Duchin, J.S., Public, H.-S., King County, E. & Team, C.C.-I. 2020 Epidemiology of Covid-19 in a Long-Term Care Facility in King County, Washington. *N Engl J Med* 382, 2005-2011. (doi:10.1056/NEJMoa2005412).
[31] Oran, D.P. & Topol, E.J. 2020 Prevalence of Asymptomatic SARS-CoV-2 Infection : A Narrative Review. *Ann Intern Med* 173, 362-367. (doi:10.7326/M20-3012).
[32] Mizumoto, K., Kagaya, K., Zarebski, A. & Chowell, G. 2020 Estimating the asymptomatic proportion of coronavirus disease 2019 (COVID-19) cases on board the Diamond Princess cruise ship, Yokohama, Japan, 2020. *Euro Surveill* 25. (doi:10.2807/1560-7917.ES.2020.25.10.2000180).
[33] Verity, R., Okell, L.C., Dorigatti, I., Winskill, P., Whittaker, C., Imai, N., Cuomo-Dannenburg, G., Thompson, H., Walker, P.G.T., Fu, H., Dighe, A., Griffin, J.T., Baguelin, M., Bhatia, S., Boonyasiri, A., Cori, A., Cucunuba, Z., FitzJohn, R., Gaythorpe, K., Green, W., Hamlet, A., Hinsley, W., Laydon, D., Nedjati-Gilani, G., Riley, S., van Elsland, S., Volz, E., Wang, H., Wang, Y., Xi, X., Donnelly, C.A., Ghani, A.C. & Ferguson, N.M. 2020 Estimates of the severity of coronavirus disease 2019: a model-based analysis. *Lancet Infect. Dis.* 20, 669-677. (doi:10.1016/S1473-3099(20)30243-7).
[34] Byambasuren, O., Cardona, M., Bell, K., Clark, J., McLaws, M.-L. & Glasziou, P. 2020 Estimating the extent of asymptomatic COVID-19 and its potential for community transmission: systematic review and meta-analysis. *medXive*. (doi:doi.org/10.1101/2020.05.10.20097543).
[35] Meyers, K.J., Jones, M.E., Goetz, I.A., Botros, F.T., Knorr, J., Manner, D.H. & Woodward, B. 2020 A cross-sectional community-based observational study of asymptomatic SARS-CoV-2 prevalence in the greater Indianapolis area. *J. Med. Virol.* 92, 2874-2879. (doi:10.1002/jmv.26182).
[36] Wang, Y., Kang, H., Liu, X. & Tong, Z. 2020 Asymptomatic cases with SARS-CoV-2 infection. *J. Med. Virol.* 92, 1401-1403. (doi:10.1002/jmv.25990).
[37] Guan, W.J., Ni, Z.Y., Hu, Y., Liang, W.H., Ou, C.Q., He, J.X., Liu, L., Shan, H., Lei, C.L., Hui, D.S.C., Du, B., Li, L.J., Zeng, G., Yuen, K.Y., Chen, R.C., Tang, C.L., Wang, T., Chen, P.Y., Xiang, J., Li, S.Y., Wang, J.L., Liang, Z.J., Peng, Y.X., Wei, L., Liu, Y., Hu, Y.H., Peng, P., Wang, J.M., Liu, J.Y., Chen, Z., Li, G., Zheng, Z.J., Qiu, S.Q., Luo, J., Ye, C.J., Zhu, S.Y., Zhong, N.S. & China Medical Treatment Expert Group for, C. 2020 Clinical Characteristics of Coronavirus Disease 2019 in China. *N Engl J Med* 382, 1708-1720. (doi:10.1056/NEJMoa2002032).
[38] Wang, Y., Wang, Y., Chen, Y. & Qin, Q. 2020 Unique epidemiological and clinical features of the emerging 2019 novel coronavirus pneumonia (COVID-19) implicate special control measures. *J. Med. Virol.* 92, 568-576. (doi:10.1002/jmv.25748).

[39] Byrne, A.W., McEvoy, D., Collins, A.B., Hunt, K., Casey, M., Barber, A., Butler, F., Griffin, J., Lane, E.A., McAloon, C., O'Brien, K., Wall, P., Walsh, K.A. & More, S.J. 2020 Inferred duration of infectious period of SARS-CoV-2: rapid scoping review and analysis of available evidence for asymptomatic and symptomatic COVID-19 cases. *BMJ Open* 10, e039856. (doi:10.1136/bmjopen-2020-039856).
[40] Larremore, D.B., Wilder, B., Lester, E., Shehata, S., Burke, J.M., Hay, J.A., Tambe, M., Mina, M.J. & Parker, R. 2020 Test sensitivity is secondary to frequency and turnaround time for COVID-19 screening. *Science Advances*, eabd5393. (doi:10.1126/sciadv.abd5393).
[41] Quilty, B.J., Clifford, S., Flasche, S., Eggo, R.M. & group, C.n.w. 2020 Effectiveness of airport screening at detecting travellers infected with novel coronavirus (2019-nCoV). *Euro Surveill* 25. (doi:10.2807/1560-7917.ES.2020.25.5.2000080).
[42] Gostic, K.M., Gomez, A.C.R., Mummah, R.O., Kucharski, A.J. & Lloyd-Smith, J.O. 2020 Estimated effectiveness of traveller screening to prevent international spread of 2019 novel coronavirus (2019-nCoV). *medRxiv*. (doi:10.1101/2020.01.28.20019224).
[43] Giri, B., Pandey, S., Shrestha, R., Pokharel, K., Ligler, F.S. & Neupane, B.B. 2020 Review of analytical performance of COVID-19 detection methods. *Anal. Bioanal. Chem.* (doi:10.1007/s00216-020-02889-x).
[44] Arevalo-Rodriguez, I., Buitrago-Garcia, D., Simancas-Racines, D., Zambrano-Achig, P., Del Campo, R., Ciapponi, A., Sued, O., Martinez-Garcia, L., Rutjes, A.W., Low, N., Bossuyt, P.M., Perez-Molina, J.A. & Zamora, J. 2020 False-negative results of initial RT-PCR assays for COVID-19: A systematic review. *PLoS ONE* 15, e0242958. (doi:10.1371/journal.pone.0242958).
